# Identification of QTLs for Resistance to *Sclerotinia sclerotiorum* in Carioca Common Bean by the *Moving Away* Method

**DOI:** 10.1155/2014/828102

**Published:** 2014-02-06

**Authors:** Letícia A. de C. Lara, João B. dos Santos, Juliana S. Veloso, Marcio Balestre, Filipe C. Alves, Monik E. Leite

**Affiliations:** ^1^Laboratório Genética Molecular, Departamento de Biologia, Universidade Federal de Lavras, Lavras, MG, Brazil; ^2^Departamento de Ciências Exatas, Universidade Federal de Lavras, Lavras, MG, Brazil

## Abstract

The aim of this study was to use multiple DNA markers for detection of QTLs related to resistance to white mold in an F_2_ population of common bean evaluated by the straw test method. The DNA from 186 F_2_ plants and from the parents was extracted for genotypic evaluation using SSR, AFLP, and SRAP markers. For phenotypic analysis, 186 F_2:4_ progenies and ten lines were evaluated, in a 14 × 14 triple lattice experimental design. The adjusted mean values of the F_2:4_ progenies were used for identification of QTLs by Bayesian shrinkage analysis. Significant differences were observed among the progenies for reaction to white mold. In identification of QTLs, 17 markers identified QTLs for resistance—13 SSRs and 4 AFLPs. The *moving away* method under the Bayesian approach proved to be efficient in the identification of QTLs when a genetic map is not used due to the low density of markers. The ME1 and BM211 markers are near the QTLs, with the effect of increasing resistance to white mold, and they have high heritability. They are thus promising for marker-assisted selection.

## 1. Introduction

Phytopathogenic organisms are the main agents responsible for significant losses in the common bean (*Phaseolus vulgaris* L.) crop, often even making the crop unviable in certain regions. Among the diseases that have most limited yield, white mold (*Sclerotinia sclerotiorum*) stands out, especially in the irrigated common bean crop [1].

It is known that the best manner of controlling most common bean diseases is the use of cultivars with some level of genetic resistance. This measure is most recommended because it avoids or reduces the use of agricultural chemicals and is economically viable for the producer. However, for control of white mold, this measure is not efficient since cultivars with a satisfactory level of resistance that are adapted to Brazilian conditions are not available. Some lines and cultivars adapted to the conditions of west-central and southeast Brazil have partial resistance [2].

To evaluate common bean resistance to white mold, there are diverse methodologies that use artificial inoculation of the pathogen [3]. Among existing methods, greatest emphasis is placed on the straw test, described by Petzoldt and Dickson [4], due to its simplicity for evaluation of physiological resistance. The straw test assists in identification, characterization, and selection of genotypes resistant to white mold, and it is the most used method in breeding programs [5].

The complexity of resistance to white mold has led many researchers to analyses of QTLs for the purpose of locating efficient molecular markers to be used in marker-assisted selection. The distribution of molecular markers throughout the genome allows for detection and localization of QTLs. Some mapping techniques have been developed, and the interval mapping method has proven to be promising. In interval mapping, the QTL genotype is not observable, but it may be predicted based on markers around it; thus, the markers define an interval that may contain a supposed QTL [6].

However, certain traits that present inheritance of the oligo- or monogenic type may show low genetic variability, and even testing a large number of markers, few polymorphic tags may be found, limiting interval analysis to preestablished linkage groups or making their construction unviable. An alternative would be the association of the marker to the phenotype, that is, where it is assumed that a marker of significant effect is in linkage disequilibrium with the QTL. Nevertheless, if this disequilibrium is unknown, the effect of the marker becomes biased and its significance confuses the effect of the marker with its frequency in recombination with the QTL [7].

An alternative is simultaneous analysis of markers and the search for QTLs in a model where the establishment of linkage groups is not necessary.

“Moving away” analysis suggested by Doerge et al. [8] allows the pivotal search for QTL using individual markers, without the need for linkage groups. This technique may be treated under the Bayesian approach, allowing the inclusion of multiple markers and analysis of multiple QTLs, which would be unviable in the approach by likelihood analysis presented by Doerge et al. [8]. Analysis of multiple QTLs is possible when one assumes, a priori, that each QTL is a random variable derived from a normal distribution with a mean of zero and individual variance, where every possible QTL is penalized through the ratio of its variance with residual variance. Thus, QTLs of small effect and low variance have their effects “shrunk” to values near zero, for they are penalized by the residual variance of the model. In contrast, QTLs of great effect tend to exhibit notable variance and are less penalized by residual variance [9]. Similar approaches were described by Xu [10] in the context of genome selection and later adapted by Wang et al. [9] for multiple interval mapping.

The aim of this study was to apply the Bayesian method of analysis by multiple markers for detection of QTLs related to white mold resistance in an F_2_ population evaluated by the straw test method.

## 2. Materials and Methods

### 2.1. Genotypes Evaluated

The lines CNFC 9506 and RP-2 were crossed. These parents were classified, based on reaction to oxalic acid, as susceptible and partially resistant to white mold, respectively, with CNFC 9506 receiving a score of 4.83 and RP-2 receiving 1.97 in the study developed by Gonçalves and Santos [2].

The CNFC 9506 line was developed by Embrapa Arroz e Feijão and the RP-2 line by UFLA, and they exhibit upright plant type and carioca (beige with brown stripes) type grains. Both are adapted and average yield (kg/ha) is greater for the RP-2 line [11].

As of the crossing of the parents, the F_1_ and F_2_ generations and the F_2:3_ and F_2:4_ progenies were obtained in field conditions. The F_2:4_ generation and ten lines (Corujinha, G122, CNFC 10720, CNFC 10722, M20, Ex-Rico 23, Small White, and Talismã) were used in the evaluation, with Corujinha being the susceptible control and Ex-Rico 23 the resistant control.

### 2.2. Evaluation of Reaction to White Mold

Evaluation of the F_2:4_ progenies was performed in the field. The experiment was conducted through a 14 × 14 triple lattice design, with a plot being represented by a one-meter row and 10 plants per plot being inoculated. A total of 196 treatments were evaluated, made up of ten lines (CNFC 9506, RP-2, Corujinha, G122, CNFC 10720, CNFC 10722, M20, Ex-Rico 23, Small White, and Talismã) and 186 progenies.

Initially sterilized sclerotia were used for obtaining the mycelium. The fungus *S. sclerotiorum* was multiplied in Petri dishes containing the potato-dextrose-agar (PDA) medium with the addition of chloramphenicol (50 mg/mL diluted in absolute alcohol) at the proportion of one drop of the antibiotic/100 mL of PDA medium and kept in BOD at 20 ± 3°C for three days and a 12-hour photoperiod. The inoculum was multiplied twice so as to obtain greater uniformity. Three days after the second multiplication, Eppendorf-type tips were used with an agar disc containing mycelium to inoculate plants at 28 days of age. For inoculation, the apex of the main plant stem was eliminated, cutting it 2.5 cm from the node, and this was placed in contact with the mycelium on the tip.

Eight days after inoculation, evaluation of resistance of the common bean to white mold was performed by means of a diagrammatic scale described by Petzoldt and Dickson [4] and modified by Singh and Terán [12].

### 2.3. Genotyping of Progenies

The DNA of the parents, CNFC 9506 and RP-2, and of the 186 F_2_ progenies, was extracted following the procedures used by Rodrigues and dos Santos [13]. The nucleic acids were rehydrated in TE buffer and quantified in 1% agarose gel using DNA markers with known concentrations. The quantified material was then diluted to 10 ng/*μ*L in pure water for PCR.

Initially, random primers of SSRs (Simple Sequence Repeats—Microsatellite), AFLPs (Amplified Fragment Length Polymorphism), and SRAPs (Sequence Related Amplified Polymorphism) were tested, and the polymorphic ones were selected, namely, 17 SSRs, 31 AFLPs, and 11 SRAPs [14]. These primers were used to genotype the 186 plants of the F_2_ population. The amplification products were subjected to vertical electrophoresis in denatured polyacrylamide gel stained in silver nitrate and photographed with a digital camera.

The genotypes of the SSR markers were identified with scores of −1, 0, and 1 for the genotypes of smallest number of base pairs, heterozygous, and genotype of greatest number of base pairs, respectively. The AFLP and SRAP markers were identified with scores 0 and 1, representing absence and presence of the band, respectively.

### 2.4. Bayesian Shrinkage Analysis

The “moving away from marker” analysis uses individual markers as a parameter in the search for QTLs. Thus, analysis is made using the conditional probabilities of the QTLs given to the reference marker. Thus, the linear model adopted is the following:

(1) $$\begin{array}{c} y_{i}=b_{0}+\sum_{j}^{m}x_{ij}a_{j}+e_{ij}, \end{array}$$

where *y*_*i*_ is the corrected mean value of the *i*th progeny *i*,  *b*_0_ is the overall mean value of the population under study, *m* is the total number of markers, *x*_*ij*_ is the genotype of the QTL, *a*_*j*_ is the effect of the QTL associated with the *j*  marker, and *e*_*ij*_ is the residue assuming *N*(0, *σ*_*e*_^2^).

In this model, it is assumed that *a*_*j*_ belongs to a normal distribution with mean value of zero and variance of *σ*_*aj*_^2^. The observable variables are the phenotypic data (*y*_*i*_) and the genotypes of the markers (*m*), while the nonobservable variables are the effects of the QTLs (*a*_*j*_), their genotypes (*x*_*ij*_), and their variances, together with the variances (*σ*_*aj*_^2^ and *σ*_*e*_^2^). A priori, it is assumed that

(2) $$\begin{array}{c} p\left(b_{0}\right)\propto 1,\;\;p\left(a_{j}\right)\propto N\left(0,\sigma_{aj}^{2}\right),\\ p\left(\sigma_{aj}^{2}\right)\propto \frac{1}{\sigma_{aj}^{2}}ep\left(\sigma_{e}^{2}\right)\propto \frac{1}{\sigma_{e}^{2}}. \end{array}$$

A priori distributions of the effects of the QTLs (*a*_*j*_), of the overall mean (*b*_0_), and of the variances (*σ*_*aj*_^2^ and *σ*_*e*_^2^) were assumed as distributions of the parameters of position and dispersion of the data by **b** and **v**, respectively, simply for ease of notation. These distributions may be described within a function of joint probability  *p*(**b**, **v**). The likelihood of the observable and nonobservable variables is given by

(3) p(y ∣ b,v)=∏i=1np(yi ∣ b,σe2)∝(σe2)−n/2  ∏i=1n×exp⁡{−12σe2∑i=1n(yi−b0−∑j=1mxijaj)2}.

In this model, only the phenotypic data are observed, whereas the genotypes of the QTLs *x*_*ij*_ are lost information that may be estimated from the *m*  markers. Taking *m*_*j*_ as the marker adjacent to a supposed QTL, we may then insert a new parameter (*λ*_*j*_), like distance, between the marker *j* and the QTL. In this study, it is assumed that each marker may be linked to a QTL, so that, a priori, it is assumed that *λ*_*j*_ is uniformly distributed between two intervals corresponding to a recombination frequency ranging from 0 (marker is the QTL itself) to 0.5 (independent segregation between the marker and the QTL):

(4) $$\begin{array}{c} p\left(b,v,\lambda \right)=p\left(b_{0}\right)p\left(\sigma_{e}^{2}\right)\prod_{k=1}^{m}p\left(\lambda_{j}\right)p\left(a_{j}\right)p\left(\sigma_{aj}^{2}\right). \end{array}$$

Assuming independence among the effects and variance and the genotypes of the QTLs, and also the independence of the observations in relation to the markers and their genetic distances, we have a new likelihood given by

(5) $$\begin{array}{c} p\left(b,v,x,\lambda \;|\;y,m\right)\propto p\left(y\;|\;b,v,x\right)p\left(x\;|\;\lambda ,m\right)p\left(b,v,\lambda \right). \end{array}$$

In F_2:4_ populations, the probability of heterozygous plants within each family is given by 0.125.

Thus, each genotype is sampled directly from a Bernoulli distribution, with probability given by

(6) p(xij ∣ λj,mj,y) =  p(xij ∣ λj,mj)p(yij ∣ b,σe2,xij)∑z=13p(xij ∣ λj,mj)p(yij ∣ b,σe2,xij).

Use of the Gibbs sampler is prohibitive because the *λ*_*j*_ parameter does not have a known function. The Gibbs sampler uses an iterative process, with a known function, taking samples from a Markov chain. When this function is unknown (not necessarily distribution a posteriori), another Markov chain Monte Carlo (MCMC) method may be used. This method is denominated Metropolis-Hastings [15, 16]. The algorithm used does not require the parameter to have a known probability. Thus, use is made of an auxiliary function that is possible to sample, taking candidate values that may be accepted with an *α* of probability.

In the method presented, a uniform distribution may be used as an auxiliary function, where  ***λ***  is sampled using the Haldane function, which is sampled over an interval delimited by max⁡⁡(0, *r*_*j*_ − *d*) and min⁡⁡(0.5, *r*_*j*_ + *d*), where  *d*  is a constant that defines the pathway within interval *j*, normally fixed between 1 and 2 cM. This function is denoted by *u*(*λ*_j_^*^, *λ*_*j*_), and the new position will be accepted in the  *k*th iteration with min⁡⁡(1, *α*) of probability, with *α* being given by [15, 16]:

(7) $$\begin{array}{c} \alpha =\;\frac{p\left(\lambda_{j}^{*}\;|\;y,b_{j},\sigma_{e}^{2}\right)u\left(\lambda_{j}^{*},\lambda_{j}\right)}{p\left(\lambda_{j}^{0}\;|\;y,b_{j},\sigma_{e}^{2}\right)u\left(\lambda_{j},\lambda_{j}^{*}\right)}. \end{array}$$

Therefore, if *α* is accepted, a new position is established and a new genotype is suggested for the  *x*  and  *w*  matrices, closing an MCMC cycle [9, 17, 18]. However, Bayesian analysis of multiple intervals has one big disadvantage. If all the possible QTLs are maintained in the model, this analysis violates, so to speak, the idea of parsimonious models. This then makes for high computational demand.

The other a posteriori conditional distributions for the  **b**  and  **v**  parameters are similar to those presented by Xu [10].

#### 2.4.1. Post-MCMC Analysis

In Bayesian inference, the significance test is not as important as in likelihood analysis. More importantly, the aim of Bayesian analysis via MCMC is to obtain an empirical a posteriori distribution from which all information in respect to the QTL may be obtained. In simple Bayesian analysis, the position of the QTL is inferred based on the number of times the effect of the QTL passes through a small region (bin) in a determined position of the genome. This curve describes the intensity profile of the QTL. In the approach of Wang et al. [9], it is assumed that each interval is associated with a QTL, so that in all the intervals, the supposed QTL will pass through all the regions of the genome, and in each interval, the same number of hits of the QTL will occur, regardless of its effect. Nevertheless, it is expected that if there is a true QTL in a given interval, its position will show a peak, whereas if the effect is null, the distribution within the interval is uniform [19]. The intensity profile of the QTL is represented by Yang and Xu [19] as a function of the *f*(*λ*) position. Nevertheless, *f*(*λ*) may not be sufficiently informative for inference concerning the QTL in Bayesian shrinkage analysis. Based on this, Yang and Xu [19] proposed the description of the effects of the QTLs according to their quadratic forms, weighed by the intensity of the position: *g*(*λ*) = *W*(*λ*)*f*(*λ*), with *W*(*λ*) = *aV*_*a*_^−1^ *a*, where *V*_*a*_^−1^ is the inverse of the variances of the effects of the QTLs given by (∑_*i*=1_^*n*^ *x*_*ij*_^2^+*σ*_*aj*_^2^/*σ*_*e*_^2^)^−1^ *σ*_*e*_^2^, which corresponds to the information matrix of the effect. This test, called the Wald test, follows a chi-square distribution with two degrees of freedom [19].

## 3. Results

**Figure 1:**
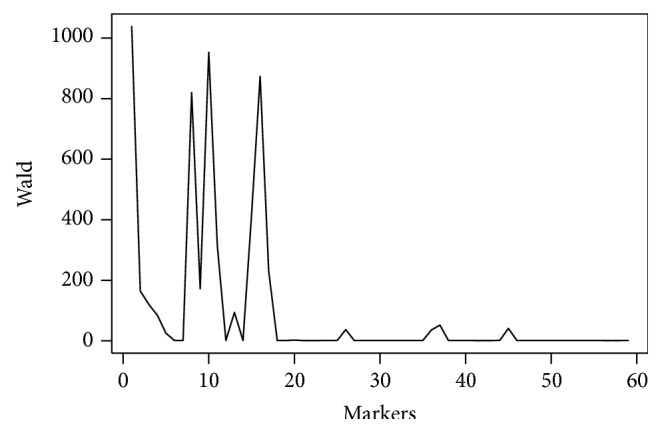
Identification of the QTLs by the Wald test.

The detection of QTLs associated with resistance to white mold through evaluation in the straw test is shown in Figure 1 and Table 1. The values of the Wald test are on the ordinate and the representation of the markers is on the abscissa, where the SSRs are markers 1 to 17, the AFLPs are markers 18 to 48, and the SRAPs are markers 49 to 59.

Among the 59 markers used, 17 identified QTLs for resistance to white mold, with 13 SSRs (BM184, BM187, BM211, BMd42a, PVM02TC116, PV188, PV74, PVESTBR_185, PVESTBR_204, PV-gaat001, ME1, BMc94, and BMc83) and four AFLPs (EAAG/MCAG_224_, EACC/MCAT_141_, EACC/MCAT_126_, and EACA/MCAT_148_). Of these, only BM184, PV188, PVESTBR_185, and BMc94 are associated with highly significant QTLs.

**Figure 2:**
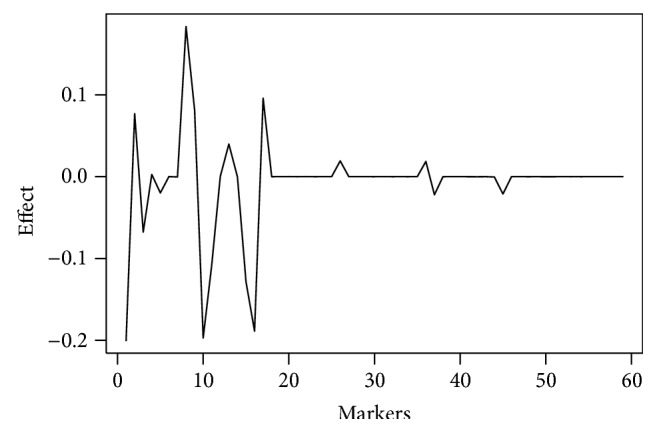
Effect of the QTL associated with the marker.

The effect of the QTLs on expression of resistance to white mold is represented in Figure 2. The effect is placed on the ordinate, ranging from −0.2 (contributes to increasing resistance) to 0.2 (contributes to reducing resistance). The representation of the markers is on the abscissa.

Among the 17 markers, nine are linked to QTLs with effects of increasing resistance to white mold. These QTLs are BM184, BM211, PVM02TC116, PVESTBR_185 PVESTBR_204, ME1, BMc94, EACC/MCAT_126_, and EACA/MCAT_148_. Of these, only three are related to highly significant QTLs (BM184, PVESTBR-185, and BMc94).

**Figure 3:**
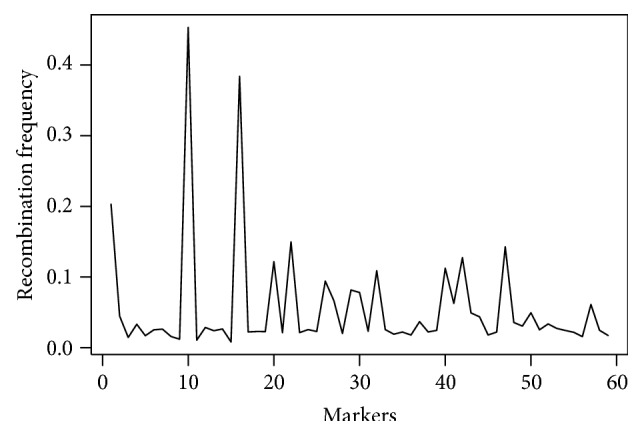
Frequency of recombination between markers and QTLs.

The frequency of recombination of the QTL with the marker is represented in Figure 3 and Table 1. The frequency of recombination is placed on the ordinate and the representation of the markers is on the abscissa.

It may be seen that the markers BM184, PVESTBR_185, and BMc94 segregate nearly independently of the QTLs associated with them, for they have a high frequency of recombination (from 20.29% to 45.31%) (Table 1, Figure 3). Therefore, they are not considered promising for marker-assisted selection (MAS).

## 4. Discussion

A summary of the distance data, in cM, between the marker and the QTL, the position of the marker in the figure, and of the effect and heritability of the QTL associated with resistance to white mold by evaluation in the straw test is shown in Table 1.

The SRAP markers were not efficient in identifying QTLs for resistance to white mold by the straw test method.

Various common bean QTLs of resistance to white mold have already been identified; however, most of them are in other countries and under environmental conditions different from the crop conditions of the southeast of Brazil [14, 20, 21]. In general, there is QTL by environment interaction and, for that reason, it is important to validate the QTLs that have already been identified, as well as to identify new QTLs in the genotypes adapted to crop conditions.

The BM211 marker was located separating two QTLs in the GL 8 [22, 23]. This linkage group contains four QTLs identified for resistance to white mold. The QTLs WM8.1^PX,GC^ and WM8.2^PX^ were first discovered by Park et al. [23] in RIL PC-50/XAN-159 populations, with an association of 9% for incidence in the field and 24% for the straw test, and later observed by Maxwell et al. [22] in RIL G122/CO72548 populations. The other two QTLs described and cited by Soule et al. [14] are WM8.3^B60,GC,BV^ and WM8.4^PR,GCmR31^. In this study, the BM211 marker is closely linked (1.45 cM) to a QTL with high heritability (76.93%). This QTL has the effect of increasing resistance to white mold; thus, it is promising for MAS.

The ME1 marker was initially marked in the GL 1 by Blair et al. [24] and afterwards was reported in the GL 9 and is present in the linkage map published by Galeano et al. [25] and Blair et al. [26]. It is also a promising marker for selection of the most resistant progenies in this study because the QTL identified has high heritability (71.37%) and is closely linked to the marker.

The BM184 marker was initially mapped in the GL 11 [24, 27]. However, Maxwell et al. [22] identified it in the GL 9, linked to the QTL WM9.1^GC^, evaluating the RIL G122/CO72548 population. This QTL confers partial resistance in the evaluation by straw test (13%) [14]. In this study, the BM184 marker identified a QTL with the effect of increasing resistance to white mold; however, it segregates almost independently from the QTL (21.53 cM). Even so, this QTL has high heritability (84.48%).

The BM187 marker was mapped in the GL 6, in which up to now only one QTL was identified for resistance to white mold, WM6.1^B60,R31^, being identified first in the RIL Benton/NY6020-4 population [28] and afterwards in the RIL Raven/I9365-31 population [14]. In this study, the BM187 marker is relatively near the QTL (4.49 cM); however, this QTL has the effect of reducing resistance to white mold and has heritability of 49.7%.

The BMd42a marker is described in the GL 10 [29, 30]. It identified a QTL of low heritability (3.01%) with the effect of reducing resistance to white mold. This marker is near the QTL, at 3.32 cM.

The SSR markers PVM02TC116, PV188, PV74, PVESTBR_185, PVESTBR_204, BMc94, and BMc83, together with the AFLP markers EAAG/MCAG_224_, EACC/MCAT_141_, EACC/MCAT_126_, and EACA/MCAT_148_, were significant in identification of QTLs of resistance to white mold; however, they have not been reported in the literature.

In this study, the ME1 and PVESTBR_204 markers are those that are closest to the QTLs—at 0.82 cM and 1.08 cM, respectively. These QTLs have the effect of increasing resistance to white mold. However, the QTL identified as PVESTBR_204 has low heritability (3.39%) and is not promising for MAS.

The PVESTBR_185 and BMc94 markers identified highly significant QTLs with high heritability (75.71% and 84.94%, resp.); however, they segregate apart from the QTL, at 75.33 cM and 50.76 cM, respectively, and they are thus not efficient for MAS.

As for the markers BM187, BMc83, and EAAG/MCAG_224_, in spite of identifying QTLs with moderate to high magnitude heritability, these QTLs have the effect of decreasing resistance to white mold. The other markers identified QTLs of low heritability and were thus not efficient.

In this study, the SRAP markers did not identify QTLs; however, their efficiency has been reported in the literature. Soule et al. [14] detected two QTLs associated with resistance to white mold in the RIL Benton/VA19 population and seven QTLs in RIL Raven/I9365-31, adding information to the genetic maps of common bean already published. One of the QTLs detected is found linked to the SCAR marker Sme1Em5, derived from a SRAP marker, and it is located in the GL 2.

## 5. Conclusions

The “moving away” method under the Bayesian approach proved to be efficient in identification of QTLs when a genetic map is not observed due to the low density of tags. In this respect, new studies may be conducted for the purpose of estimating the position and order of the QTLs in the genome using a consensus map.

The ME1 and BM211 markers are near the QTLs, with the effect of increasing resistance to white mold and they are of high heritability; they are therefore promising for marker-assisted selection in progenies derived from cultivars adapted to the conditions of the southeast of Brazil.

## Figures and Tables

**Table 1:** Summary of the distance between the marker and QTL, position of the markers in Figures 1, 2, and 3, effect of the QTL associated with the marker, and its respective magnitudes and heritability of the QTL.

Marker	Position	FR^a^ (%)	Distance^b^	Effect	Wald^c^	Herit^d^ (%)
BM184	1	20.29	21.53	0.2	1038.04	84.48
BM187	2	4.47	4.49	−0.077	164.57	49.7
BM211	3	1.45	1.45	0.068	120.37	76.93
BMd42a	4	3.32	3.32	−0.003	84.46	3.01
PVM02TC116	5	1.7	1.7	0.02	25.24	2.57
PV188	8	1.59	1.59	−0.184	819.59	3.74
PV74	9	1.21	1.21	−0.081	171.98	0.87
PVESTBR_185	10	45.32	75.33	0.197	952.96	75.71
PVESTBR_204	11	1.08	1.08	0.108	313.72	3.39
PV-gaat001	13	2.41	2.41	−0.04	93.98	0.12
ME1	15	0.82	0.82	0.129	416.94	71.37
BMc94	16	38.39	50.76	0.189	873.32	84.94
BMc83	17	2.24	2.24	−0.096	230.64	57.92
EAAG/MCAG_224_	26	9.42	9.54	−0.019	37.03	77.51
EACC/MCAT_141_	36	1.8	1.8	−0.019	35.74	0.39
EACC/MCAT_126_	37	3.68	3.69	0.022	52.11	2.01
EACA/MCAT_148_	45	1.8	1.8	0.021	41.08	4.92

^a^Frequency of recombination; ^b^distance, in cM, from the marker to the QTL; ^c^value of the Wald test; ^d^heritability of the QTL; +: increased resistance; −: decreased resistance.
